# Pharmacological Dissection of the Crosstalk between Na_V_ and Ca_V_ Channels in GH3b6 Cells

**DOI:** 10.3390/ijms23020827

**Published:** 2022-01-13

**Authors:** Léa Réthoré, Joohee Park, Jérôme Montnach, Sébastien Nicolas, Joseph Khoury, Elodie Le Seac’h, Kamel Mabrouk, Harold De Pomyers, Hélène Tricoire-Leignel, César Mattei, Daniel Henrion, Ziad Fajloun, Michel De Waard, Claire Legendre, Christian Legros

**Affiliations:** 1 INSERM, CNRS, MITOVASC, Equipe CarME, SFR ICAT, University of Angers, 49000 Angers, France; lea.rethore@univ-angers.fr (L.R.); pakjoohee25@gmail.com (J.P.); joseph-0-khoury@hotmail.com (J.K.); elodieleseach@gmail.com (E.L.S.); helene.tricoire-leignel@univ-angers.fr (H.T.-L.); cesar.mattei@univ-angers.fr (C.M.); daniel.henrion@univ-angers.fr (D.H.); 2 Institut du Thorax, Université de Nantes INSERM UMR 1087-CNRS UMR 6291, 44007 Nantes, France; jerome.montnach@univ-nantes.fr (J.M.); sebastien.nicolas@univ-nantes.fr (S.N.); michel.dewaard@univ-nantes.fr (M.D.W.); 3 Department of Biology, Faculty of Science III, Michel Slayman Tripoli Campus, Lebanese University, Tripoli 1352, Lebanon; zfajloun@gmail.com; 4 Institut de Chimie Radicalaire, UMR CNRS 7273, Aix-Marseille Université, 13013 Marseille, France; kamel.mabrouk@univ-amu.fr; 5 Latoxan Laboratory, 26800 Portes lès Valence, France; harold.pomyers@latoxan.com; 6 Laboratory of Applied Biotechnology (LBA3B), Azm Centre for Research in Biotechnology and Its Application, EDST, Lebanese University, Tripoli 1300, Lebanon; 7 Laboratory of Excellence Ion Channels, Science & Therapeutics, 59650 Villeneuve d’Ascq, France

**Keywords:** GH3b6 cells, voltage-gated sodium channel, L-type voltage-gated calcium channel, veratridine, patch-clamp, Fura-2

## Abstract

Thanks to the crosstalk between Na^+^ and Ca^2+^ channels, Na^+^ and Ca^2+^ homeostasis interplay in so-called excitable cells enables the generation of action potential in response to electrical stimulation. Here, we investigated the impact of persistent activation of voltage-gated Na^+^ (Na_V_) channels by neurotoxins, such as veratridine (VTD), on intracellular Ca^2+^ concentration ([Ca^2+^]_i_) in a model of excitable cells, the rat pituitary GH3b6 cells, in order to identify the molecular actors involved in Na^+^-Ca^2+^ homeostasis crosstalk. By combining RT-qPCR, immunoblotting, immunocytochemistry, and patch-clamp techniques, we showed that GH3b6 cells predominantly express the Na_V_1.3 channel subtype, which likely endorses their voltage-activated Na^+^ currents. Notably, these Na^+^ currents were blocked by ICA-121431 and activated by the β-scorpion toxin Tf2, two selective Na_V_1.3 channel ligands. Using Fura-2, we showed that VTD induced a [Ca^2+^]_i_ increase. This effect was suppressed by the selective Na_V_ channel blocker tetrodotoxin, as well by the selective L-type Ca_V_ channel (LTCC) blocker nifedipine. We also evidenced that crobenetine, a Na_V_ channel blocker, abolished VTD-induced [Ca^2+^]_i_ elevation, while it had no effects on LTCC. Altogether, our findings highlight a crosstalk between Na_V_ and LTCC in GH3b6 cells, providing a new insight into the mode of action of neurotoxins.

## 1. Introduction

Voltage-gated Na^+^ (Na_V_) channels are key molecular components involved in the electrical-excitability properties of the so-called excitable cells, such as neurons and myocytes (i.e., they can develop action potentials in response to electrical stimulation) [1]. Na_V_ channels constitute validated pharmacological molecular targets for a large panel of clinically used drugs, such as anti-arrhythmics, anti-convulsants, anesthetics, and analgesics [2]. They are also targeted by various natural toxins from animals, plants, and microorganisms [2,3]. In mammalian genomes, nine genes (*scn1a*, *2a*, *3a*, *4a*, *5a,* and *scn8a*, *9a*, *10a*, *11a*) encode as many Na_V_ channel α-subunit isoforms (Na_V_1.1, 1.2, 1.3, 1.4, 1.5, 1.6, 1.7, 1.8, and 1.9) [4,5]. These genes share high sequence identities and pharmacological properties among mammalian species, in particular in rodents and humans [5]. Na_V_ channels are pharmacologically classified according to their sensitivity to tetrodotoxin (TTX). Six isoforms are highly sensitive (nanomolar range) to TTX (TTX-S): Na_V_1.1, 1.2, 1.3, 1.4, Na_V_1.6, and Na_V_1.7 and reciprocally three isoforms are much less sensitive and thus called resistant to TTX (TTX-R): Na_V_1.5, 1.8, and Na_V_1.9 [2,3]. These α-subunits are complex pore-forming and glycosylated membrane proteins containing all molecular determinants needed to form a rapid inactivating voltage-gated channel, highly selective to Na^+^ [5]. They are associated with one or two auxiliary β-subunits (Na_V_β1–4), encoded by four different genes. These β-subunits play a chaperon, gating, and regulatory role for Na_V_ channels and belong to the cell adhesion molecule family [6].

The pharmacology of Na_V_ channels is particularly vast and complex. At least seven molecular binding sites (Sites 1–7) have been described for various neurotoxins, pyrethroids, local anesthetics, antiarrhythmics, and antiepileptics [2,7,8]. All clinically used drugs block Na_V_ channels through binding to the so-called “local anesthetic receptor site”, within the pore. TTX and saxitoxin, two natural alkaloids, are pore-binding blockers, defining Site 1. Various neurotoxins act as activators, such as (i) veratridine (VTD) and batrachotoxin (BTX) through binding to Site 2; (ii) animal toxins (from scorpion, snake, wasp and sea anemone), which interact with Sites 3 and 4; (iii) polycylic toxins including brevetoxins (with PbTx-2) and ciguatoxins, which interact with Site 5; (iv) δ-conotoxins by binding to Site 6; and (v) pyrethroid insecticides with delthamethrin, defining Site 7 [2,7,8].

Na_V_ channels are mainly expressed in excitable cells, such as neurons of the central nervous system (Na_V_1.1–3 and Na_V_1.6) and of the peripheral nervous system (Na_V_1.6, 1.7, 1.8, 1.9 and possibly 1.1), myocytes (Na_V_1.4), and cardiomyocytes (Na_V_1.5) [2]. In neurons [9,10,11,12], synaptosomes [13], and neuroblastoma cells [14,15,16,17], Na_V_ channels could be activated by neurotoxins, such as VTD or brevetoxin (PbTx), leading to a large intracellular Ca^2+^ overload, as a consequence of the activation of Na^+^/Ca^2+^ exchanger reverse mode (NCX), or/and NMDA receptor or/and voltage-gated Ca^2+^ channels (Ca_V_), including L-type Ca_V_ (LTCC). Besides the neurotoxicity linked to Ca^2+^ overload, these mechanisms reveal a close relationship between Na^+^ and Ca^2+^ homeostasis, which is crucial for the regulation of membrane excitability [11,18,19].

Na_V_ channels are also expressed in endocrine networks, such as chromaffin cells, pancreatic β cells, and somatotropic cells in the pituitary gland, which also exhibit membrane excitability properties playing a key role in hormone release [18,20,21]. Among endocrine cells, the GH3 pituitary cell line and subclones, such as GH4C1 and GH3b6 cells, exhibit spontaneous action potentials and intracellular Ca^2+^ oscillations that can be modified by altering Na_V_ channel activity [22,23]. Interestingly, these characteristics have been already used to test the effects of new toxins and drugs targeting Na_V_ channels using electrophysiology [24,25,26] but never for a cell-based assay using fluorescent probe.

Here, we hypothesized that activation by neurotoxins of Na_V_ channels endogenously expressed in GH3b6 cells would lead to an increase of the intracellular Ca^2+^ concentration ([Ca^2+^]_i_), which could be measured by a Ca^2+^ fluorescent probe. By combining RT-qPCR, immunoblotting and immunolocalization experiments, we showed for the first time that GH3b6 cells mainly express the Na_V_1.3 channel subtype and the Na_V_β1 subunit. The gating and pharmacological properties of Na^+^ currents elicited by these cells indeed correspond to Na_V_1.3 channels. We demonstrated that the pharmacological activation of Na_V_ channels induces [Ca^2+^]_i_ elevation mediated by LTCC. Taken together, our data highlight a crosstalk between Ca^2+^ and Na^+^ homeostasis in GH3b6 cells and particularly between Na_V_1.3 and LTCC.

## 2. Results

### 2.1. GH3b6 Cells Mainly Express the Na_V_1.3 Channel Subtype

**Figure 1:**
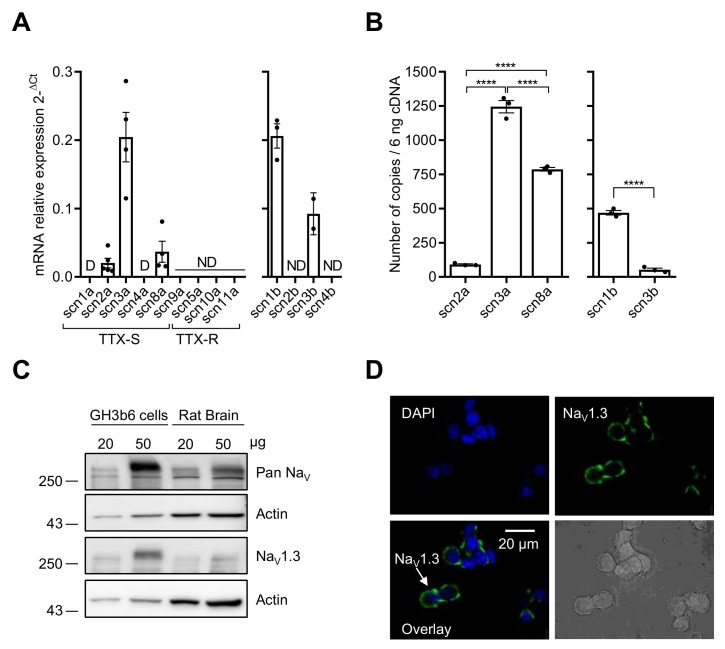
Na_V_ channel expression in GH3b6 cells. (**A**) The mRNA expression levels of all α and β subunits were first determined by relative RT-qPCR. (**B**) RT-qPCR with absolute quantification for the genes, which were detected by relative RT-qPCR. One-way ANOVA (**** *p* < 0.0001) followed by Tukey post-hoc multiple comparison test was performed. The data are mean ± SEM. D: Disregarded (Ct > 32); ND: Not Detected. (**C**) Western blot analysis of Na_V_ channel expression. Immunoblotting was performed using pan-Na_V_ and Nav1.3 channels antibodies with 20 and 50 µg of protein extracts from GH3b6 cells and rat brain, after separation on 8% SDS-PAGE. Actin was the loading control. (**D**) Immunocytolocalization of Na_V_1.3 channels in GH3b6 cells. Fluorescence labeling (green) using Alexa Fluor 488 anti-mouse secondary antibody allowed the detection of Na_V_1.3 channels at the plasma membrane. Nuclei (blue) were stained with DAPI. Original magnification ×60.

We first characterized the expression of Na_V_ channels in GH3b6 cells by combining RT-qPCR, immunoblotting, and immunolocalization (Figure 1). *Scn2a*, *scn3a*, and *scn8a* cDNAs encoding three TTX-S Na_V_ channels (Na_V_1.2, Na_V_1.3, and Na_V_1.6, respectively) were amplified (Figure 1A). *Scn1a* and *scn4a* cDNAs encoding Na_V_1.1 and Na_V_1.4 were also detected but at very low levels (Ct-values > 32, with 10 ng of cDNA), and thus their expression was disregarded. Absolute quantification of *scn2a*, *scn3a*, and *scn8a* mRNA copies showed that the transcription level of *scn3a* was about 13.7-fold and 1.6-fold higher than those of *scn2a* and *scn8a*, respectively (*p* < 0.0001, *n* = 3; Figure 1B). RT-qPCR experiments allowed the amplifications of *scn1b* and *scn3b* cDNAs encoding Na_V_β1 and Na_V_β3 subunits while *scn2b* and *scn4b* cDNAs were not detected (Figure 1A). The number of *scn1b* mRNA copies was 8.9-fold higher than that of *scn3b* (*p* < 0.0001, *n* = 3, Figure 1B). 

Since the detection of mRNAs encoding these Na_V_ channel subtypes does not mandatorily reflect their expression at the protein level, we performed Western blot and immunocytochemistry analysis. The immunoblots of GH3b6 cell protein extracts showed an intense immunoreactivity for a band with an apparent molecular weight of ~250 kDa with Pan-Na_V_ and Na_V_1.3 antibodies (Figure 1C). While Na_V_1.2 were immunodetected using protein extracts from rat brain, the Western blot with GH3b6 cell proteins did not show Na_V_1.2 channels at the protein level (Supplementary Figure S1). In addition, the antibodies against Na_V_1.6 allowed strong immunofluorescent labeling with neurons but not with GH3b6 cells (Supplementary Figure S2). The expression of the Na_V_1.3 channel at the plasma membrane was confirmed by fluorescent labeling with a specific monoclonal antibody (Figure 1D). Altogether, these findings showed that the Na_V_1.3 channel is the main Na_V_ channel subtype expressed at the protein level in GH3b6 cells. 

**Figure 2:**
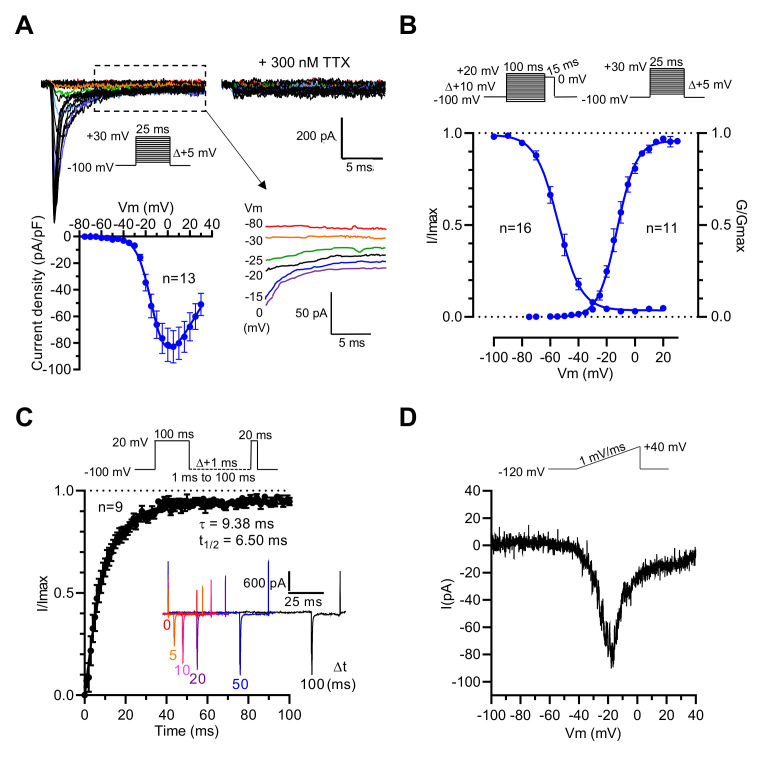
Electrophysiological characterization of I_Na_ in GH3b6 cells by manual patch-clamp recording. (**A**) Representative family of Na^+^ current traces recorded in GH3b6 cells before and after the application of 300 nM TTX. The currents were elicited by stepping the membrane potential (Vm) as shown in inset. I–V relationship curve obtained by plotting the mean peak current density to Vm (*n* = 13). Data were fitted to the equation of Stuehmer with E_Na_ = 58.2 ± 5.1 mV, g = 1.92 ± 1.34 nS, V_1/2_ = −10.39 ± 0.97 mV, and k = 7.06 ± 0.33 (*n* = 13). Selected traces illustrating persistent currents are shown in an enlarged view. (**B**) The voltage dependences of activation (circles) and inactivation (close circles). Depolarization protocols are shown in insets. Fitting was done with the Boltzmann equation as described in the “Material and Methods” section. (**C**) The graph shows the kinetic of recovery from inactivation at −100 mV. The data (mean ± SEM) were best fitted with a monoexponential equation. To illustrate the rate of recovery from inactivation, selected traces are shown in the inset. (**D**) Example of the ramp response. The current evoked during increasing the voltage ramp from −120 mV to + 40 mV during 160 ms is shown.

Thus, it is likely that the Na_V_1.3 channel subtype endorses the genesis of voltage-activated Na^+^ current in GH3b6 cells. To address this hypothesis, we characterized the Na^+^ current (I_Na_) in GH3b6 cells by patch-clamp electrophysiology. As expected, depolarizing pulses triggered inward currents, which were blocked by a low concentration of TTX (300 nM, Figure 2A), in accordance with the presence of TTX-S Na_V_ channels as previously described in GH3 cells [27,28]. Current–voltage relationships showed that I_Na_ activated between −45 and −40 mV, gradually increased to a maximum current density of −82.87 ± 12.17 pA/pF at −5 mV, and reversed at +58.20 ± 5.14 mV (Figure 2A). We also observed persistent currents at the end of the depolarization pulse (Figure 2A). The voltage dependence of the activation and steady-state inactivation of I_Na_ were assessed using specific voltage-clamp protocols. The normalized conductance or peak current amplitudes were plotted versus voltage and fitted to the Boltzmann equation, yielding a V_1/2_ of −12.23 ± 1.34 mV (*n* = 11) for activation, and a V_1/2_ of −53.58 ± 1.66 (*n* = 16) for inactivation (Figure 2B, Table 1). The recovery from inactivation was also examined and the analysis of the data showed that the recovery of I_Na_ is mono-exponential with a time constant of 9.38 ms. These data were in agreement with those previously reported [27,29]. Finally, to confirm that I_Na_ in GH3b6 cells are endorsed by Na_V_1.3 channels, we tested slow ramp depolarization stimulation (Figure 2D). Our data showed that GH3b6 cells indeed produce a ramp-triggered inward current, which is the hallmark of the Na_V_1.3 channel [30]. 

**Figure 3:**
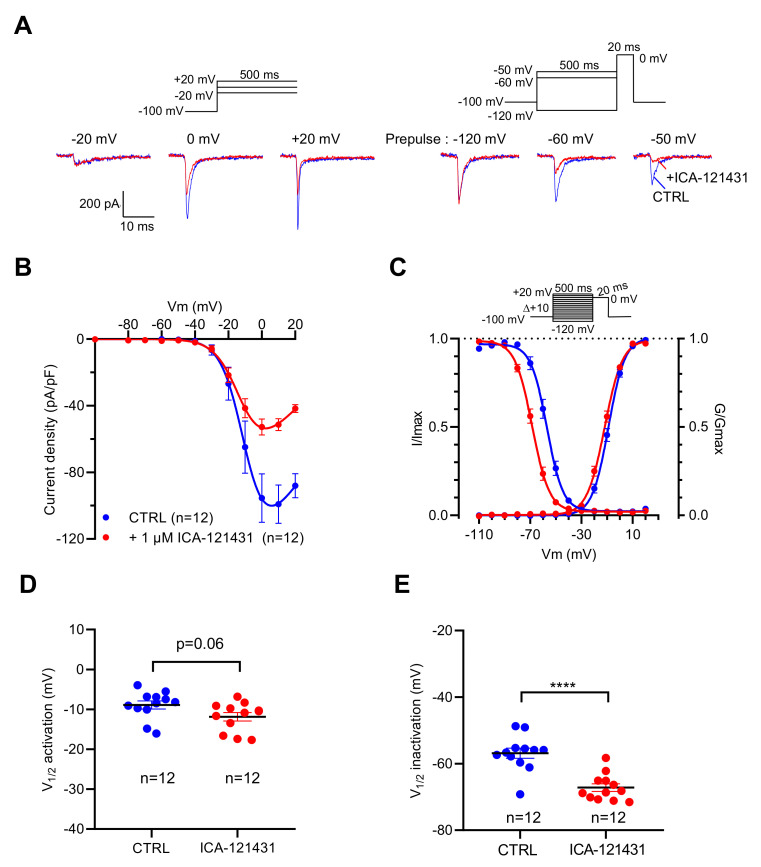
Effects of ICA-121431, a selective inhibitor of Na_V_1.3 channels, on I_Na_ recorded in GH3b6 cells by manual patch-clamp. (**A**,**B**) To illustrate the effects of ICA-121431, examples of superimposed I_Na_ elicited by 500 ms depolarizing pulses (at −20, 0, and +20 mV) and examples of superimposed I_Na_ elicited by depolarizing pulse at 0 mV immediately after a 500 ms prepulse (−120, −60, and −50 mV) are shown. The control traces are in blue (CTRL) and red traces correspond to I_Na_ after ICA-121431 application (1 µM). (**B**) Current–voltage relationships and (**C**) activation/inactivation curves of I_Na_ recorded before (blue circles) and after 1 µM ICA-121431 (red circles). Comparison of V_1/2_ activation (**D**) and inactivation (**E**) in the absence (CTRL) and in the presence of 1 nM Tf2. Statistical analysis was performed using the two-tailed unpaired *t*-test, **** *p* < 0.0001. Data represent the mean ± SEM.

To explore the contribution of the Na_V_1.3 channel subtype in the genesis of I_Na_ in this cell line, we used ICA-121431 [31,32,33] and the β-scorpion toxin Tf2 [32,34], two selective ligands of this Na_V_ channel subtype. At 1 µM, ICA-121431 reduced by 50% the amplitude of I_Na_ when they were elicited by depolarizing pulse from −100 mV, while it almost abolished I_Na_ evoked by the depolarizing pulse from −50 mV (Figure 3A). ICA-121431 reduced the I_Na_ amplitude in a non-voltage-dependent manner (Figure 3B), and thus did not alter the voltage dependency of activation (*p* = 0.06) (Figure 3C,D). However, ICA-121431 induced a negative shift of 10.3 mV (*p* < 0.0001) of the voltage dependence of inactivation (Figure 3C,E). These data are in agreement with previous data indicating that ICA-121431 preferentially interacts with inactivated Na_V_1.3 channels [31,32,33]. At a very low concentration (1 nM), Tf2 strongly altered the activation of I_Na_. 

**Figure 4:**
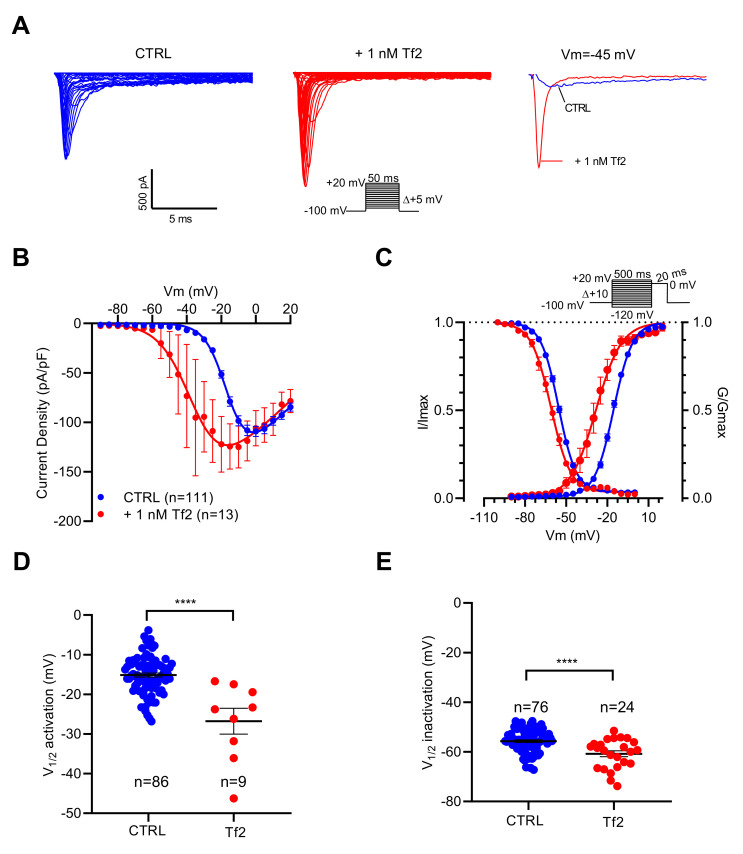
Effects of Tf2, a selective β-scorpion toxin of Na_V_1.3 channels, on I_Na_ recorded in GH3b6 cells by automated patch-clamp. (**A**) Representative examples of I_Na_ elicited by 50 ms depolarization steps (protocol inset), in the absence and presence of 1 nM Tf2. To illustrate the strong effect of 1 nM Tf2, superimposed I_Na_ traces at −45 mV are shown. (**B**) Current–voltage relationships (**C**) and activation/inactivation curves of I_Na_ recorded before (blue circles) and after 1 nM Tf2 (red circles). Comparison of V_1/2_ activation (**D**) and inactivation (**E**) in the absence (CTRL) and in the presence of 1 nM Tf2. Statistical analysis was performed using the two-tailed unpaired *t*-test, **** *p* < 0.0001. Data represent the mean ± SEM.

As illustrated in Figure 4A, depolarizing pulses elicited I_Na_ with higher amplitudes in the presence of the Tf2 compared to the control. The current–voltage relationships showed that Na_V_ channels opened at potentials more negative in the presence than in the absence of Tf2 (Figure 4B). The V_1/2_ of activation was positively shifted by 11.7 mV (*p* < 0.0001) (Figure 4C,D). In addition, Tf2 induced a negative shift of V_1/2_ of inactivation by 5.1 mV (*p* < 0.0001) (Figure 4C,E). In conclusion, I_Na_ is sensitive to both ICA-121431 and Tf2, indicating that it is promoted by the Na_V_1.3 channel subtype.

### 2.2. Activation of Na_V_ Channels by Various Neurotoxins Triggers the Increase of Intracellular Ca^2+^ in GH3b6 Cells 

**Figure 5:**
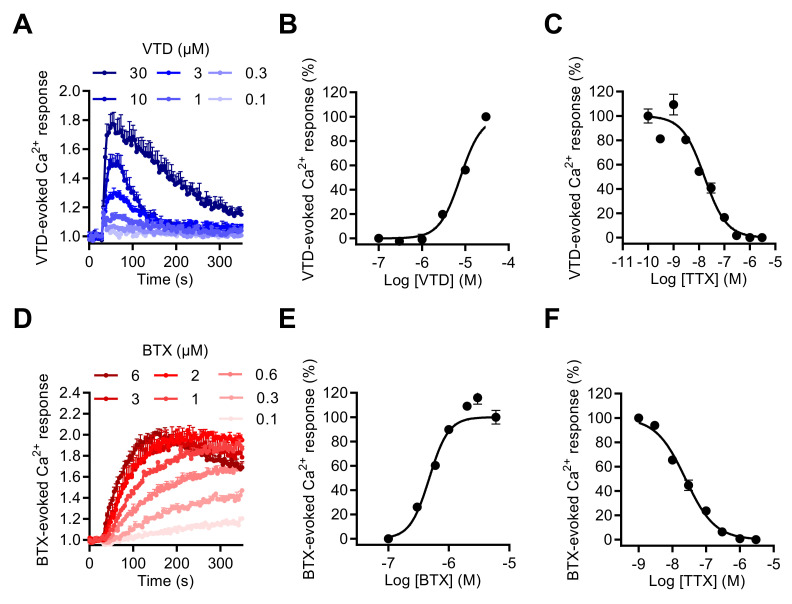
Effects of VTD and BTD, two selective Na_V_ channel activators, on the intracellular Ca^2+^ level in GH3b6 cells. (**A**,**D**) Representative kinetics of Fura-2 fluorescence in GH3b6 cells treated with increasing concentrations of VTD or BTX. The increase of the Fura-2 fluorescence emission ratio reflects [Ca^2+^]_i_ elevation. (**B**,**E**) The Ca^2+^ responses induced by VTD and BTX are concentration dependent. In both cases, the concentration–response relationships were analyzed using the Hill–Langmuir equation with variable slope. The values of EC_50_ and the Hill coefficient were respectively 5.39 ± 2.3 µM and 2.00 ± 0.21 with VTD (R^2^ = 0.98) and 0.66 ± 0.18 µM, 2.88 ± 0.15 with BTX (R^2^ = 0.95). (**C,F**) The Na_V_ channels involved in Ca^2+^ responses induced by VTD and BTX are TTX-S. TTX inhibits in a concentration-dependent manner the Ca^2+^ responses elicited by 10 µM of VTD (**C**) or by 1 µM of BTX (**F**) with similar IC_50_ (15.63 ± 0.72 nM, 21.13 ± 3.32 nM, respectively) and Hill coefficients (1.21 ± 0.21, R^2^ = 0.97 and 0.95 ± 0.04, R^2^ = 0.99 for VTD and BTX, respectively). Data represent the mean ± SEM (*n* = 3 wells) and are representative of at least two independent experiments.

To explore the impact of Na_V_ channel activation by neurotoxins on [Ca^2+^]_i_, we first determined whether the two most often used Na_V_ neurotoxins, VTD and BTX, could induce [Ca^2+^]_i_ elevation in GH3b6 cells. Using the Ca^+^ fluorescent probe, Fura 2-AM, we showed that both VTD and BTX efficiently increased Fura-2 fluorescence, which indicated [Ca^2+^]_i_ elevation (Figure 5A,D). Thus, both toxins were able to induce [Ca^2+^]_i_ elevation with different kinetics and in a TTX-sensitive manner (Figure 5A,D). Indeed, the BTX-induced Ca^2+^ response was slow and reached a plateau after 250 s of injection, while in comparison, VTD induced a transient increases in [Ca^2+^]_i_, which peaked at ~1 min. The VTD and BTX-induced Ca^2+^ responses were concentration dependent and best fitted by the Hill–Langmuir equation for a bimolecular reaction (Figure 5B,E). As expected, VTD exhibited a lower potency, with an EC_50_ of 5.39 ± 2.3 µM (Figure 5B), compared to BTX, with an EC_50_ of 0.66 ± 0.18 µM (Figure 4E). The TTX-induced inhibition of VTD- and BTX-evoked Ca^2+^ responses was fitted by a single site bimolecular equation, yielding similar IC_50_ values in the nanomolar range in each case (IC_50_ of 15.63 ± 0.72 nM with VTD and IC_50_ of 21.13 ± 3.32 nM with BTX), in agreement with the blockade of TTX-S Na_V_1.3 channels (Figure 5C,F).

**Figure 6:**
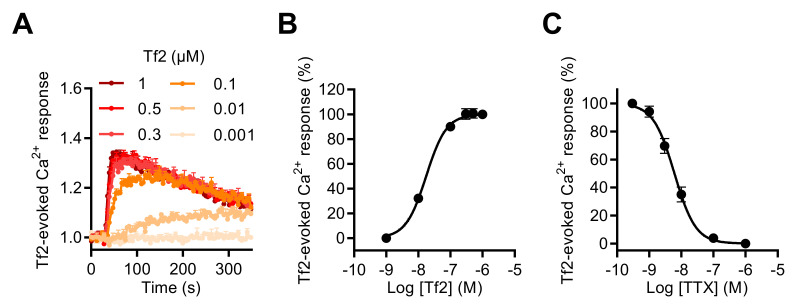
Effect of Tf2, a selective β-scorpion toxin of Na_V_1.3, on the intracellular Ca^2+^ level in GH3b6 cells. (**A**) Representative kinetics of Fura-2 fluorescence emission in GH3b6 cells treated with increasing concentrations of Tf2. (**B**) The Ca^2+^ responses induced by Tf2 are concentration dependent. The concentration–response relationships were analyzed using the Hill–Langmuir equation with variable slope. The values of EC_50_ and the Hill coefficient were 17.51 ± 1.4 nM and 1.378 (R^2^ = 0.99). (**C**) TTX inhibits in a concentration-dependent manner the Ca^2+^ responses elicited by 0.5 µM of Tf2 with an IC_50_ value of 7.9 ± 2.1 nM and a Hill coefficient of 1.31 ± 0.135, R^2^ = 0.94. The data represent the mean ± SEM (*n* = 3 wells) and are representative of at least two independent experiments.

To investigate the involvement of the Na_V_1.3 subtype in the [Ca^2+^]_i_ increase induced by Na_V_ channel neurotoxins in GH3b6 cells, we used, as in the patch-clamp recordings of I_Na_, the Na_V_1.3-selective scorpion toxin, Tf2. This toxin also induced [Ca^2+^]_i_ elevation in our cell model and the Tf2-induced Ca^2+^ responses were concentration dependent with an EC_50_ value of 17.51 ± 1.4 nM and best fitted by the Hill–Langmuir equation for a bimolecular reaction with a Hill slope of 1.38 (Figure 6A,B). The TTX inhibition of Tf2-evoked Ca^2+^ responses was fitted by a single site bimolecular equation, giving an IC_50_ value of 7.9 ± 2.1 nM and a Hill coefficient of 1.31 ± 0.14, in agreement with the blockade of the TTX-S Na_V_1.3 channel subtype (Figure 6C). 

**Figure 7:**
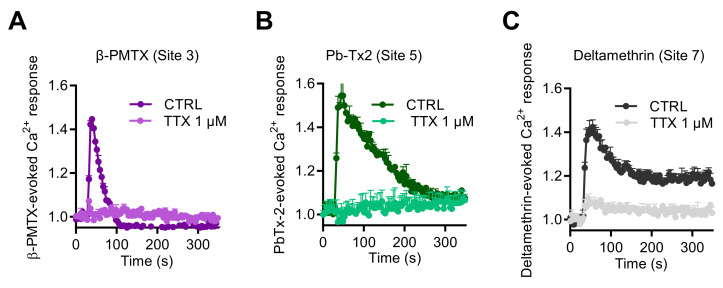
Effects of Na_V_ channel activators, which bind to Site 3, Site 5, and Site 7, on intracellular Ca^2+^ in GH3b6 cells. Representative Fura-2 fluorescence kinetics traces were obtained after injection of 100 µM β-PMTX (**A**), 3 µM of Pb-Tx2 (**B**), or 10 µM of deltamethrine (**C**). All of them were fully abolished by TTX use at 1 µM. Data represent the mean ± SEM (*n* = 3 wells) from at least two independent experiments.

Finally, to explore if other neurotoxins, which activate Na_V_ channels through binding to other pharmacological sites, could also enhance [Ca^2+^]_i_, we tested the wasp venom peptide β-PMTX (Site 3) [31,35,36], the marine toxin PbTx-2 (Site 5), and the pyrethroid deltamethrine (Site 7). These three molecules induced intracellular Ca^2+^ responses with various kinetic profiles (Figure 7). At 100 µM, β-PMTX triggered a rapid and transient Ca^2+^ elevation, in a concentration-dependent manner, as observed with VTD (Figure 7A). In comparison, PbTx-2 (3 µM) provoked a rapid response followed by a slower decrease of the Ca^2+^ signal (Figure 7B). In contrast, deltamethrine (10 µM) caused Ca^2+^ responses with a peak, followed by a plateau (Figure 7C). Ca^2+^ responses induced by these three toxins were fully blocked by TTX at 1 µM.

### 2.3. Na_V_ Channel Activation by Neurotoxins Triggers Ca^2+^ Influx Mediated by L-Type Ca_V_ Channels (LTCC)

Since it is well-known that in neurons, persistent Na^+^ entry induced by VTD is associated with [Ca^2+^]_i_ elevation mediated by the Na^+^/Ca^2+^ exchanger NCX, and/or LTCC, and/or N-type channels [11,13,15,17,37], we challenged whether NCX and Ca_V_ channels contribute to VTD-induced Ca2+ responses. To identify the source of Ca^2+^ mobilized by VTD, Fura-2 experiments were performed in Ca^2+^-free medium. In this condition, the VTD-evoked Ca^2+^ response was completely suppressed (Supplementary Figure S3). Moreover, the depletion of Ca^2+^ stores by with thapsigargin (2 µM, 10 min prior to injection of VTD) did not modify the VTD-induced Ca^2+^-response (Supplementary Figure S3). Thus, Na_V_ channel activation by VTD did not involve intracellular Ca^2+^ stores and leads to Ca^2+^ entry mediated by transport through the plasma membrane by NCX and/or Ca_V_ channels.

**Figure 8:**
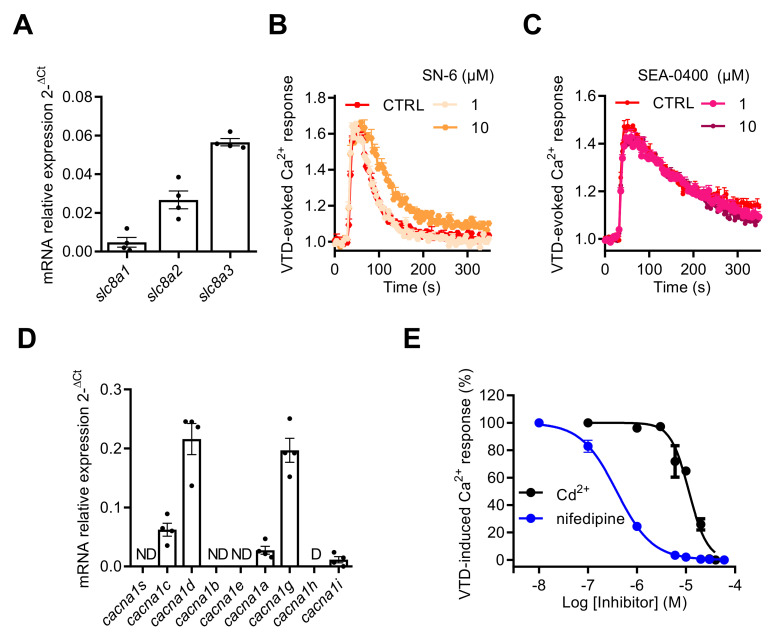
Effects of NCX and Ca_V_ channel inhibitors on VTD-evoked Ca^2+^ responses in GH3b6 cells. (**A**) The expression profile of NCX1–3 genes (*scl8a1–3*) in GH3b6 cells was characterized by relative RT-qPCR. The data are mean of mRNA relative expression ± SEM. Effects of SN-6 (**B**) and SEA0400 (**C**), two specific inhibitors of NCX, on VTD-induced Ca^2+^ responses in GH3b6 cells. VTD was used at 10 µM. (**D**) The expression profile of Ca_V_ channel genes (*cacna1a–e*, *g–i*, and *s*) in GH3b6 cells was characterized by relative RT-qPCR. The data are mean ± SEM. D: Disregarded (Ct > 32); ND: Not Detected. (**E**) Concentration–inhibition relationship of VTD-induced Ca^2+^ responses by nifedipine, a specific blocker of L-type Ca_V_ channels (IC_50_ = 1.04 ± 0.65 µM, Hill slope = 1.30 ± 0.11, R^2^ = 0.98) and Cd^2+^, a blocker of Ca_V_ channels (IC_50_ = 9.76 ± 1.74 µM, Hill slope = 2.35 ± 0.03, R^2^ = 0.97) with VTD used at 10 µM. Data represent the mean ± SEM (*n* = 3) from two independent experiments.

The NCX gene expression profile by RT-qPCR revealed that NCX1–3 subtype transcripts were expressed in GH3b6 cells with the following ranking order: *slc8a3* > *slc8a2* > *slc8a1* (Figure 8A). Since KB-R7943 inhibited the Na^+^ current [38], we used the two other NCX inhibitors available: the selective NCX inhibitor SN-6 [39] with IC_50_ values of 2.9, 16, and 8.6 µM for NCX1, NCX2, and NCX3, respectively, and the selective NCX1 and NCX2 inhibitor SEA-0400 [40] with IC_50_ values of 0.056 and 0.98 µM, respectively. Both SN-6 and SEA-0400 did not significantly inhibit the VTD-induced Ca^2+^ responses, at a concentration of 10 µM (Figure 8B,C), thus excluding the implication of NCX. Next, we characterized the gene expression profiles of Ca_V_ channels by RT-qPCR in GH3b6 cells. The cDNAs of *cacna1c* and *cacna1d*, encoding two LTCC (Ca_V_1.2 and Ca_V_1.3), *cacna1a* encoding a P/Q type Ca_V_ channel (Ca_V_2.1), *cacna1g* and *cacna1i*, encoding two t-type Ca_V_ channels (Ca_V_3.1 and Ca_V_3.3) (Figure 8D), were detected at significant expression levels. The highest expression levels of transcription were found for *cacna1c*, *cacna1d*, and *cacna1i*, indicating that both L-type and T-type Ca_V_ channels represent the main Ca_V_ channel subtypes expressed in GH3b6 cells, as previously described in GH3 or GH3b6 cells [41,42]. The VTD-evoked Ca^2+^ responses were totally suppressed either by Cd^2+^ (at ~30 µM) or nifedipine (at ~10 µM) (Figure 8E). Both compounds induced a concentration-dependent inhibition of VTD-evoked Ca^2+^ responses, which were best fitted with the Langmuir–Hill equation, with IC_50_ values of 1.04 ± 0.65 µM for nifedipine and 9.76 ± 1.74 µM for Cd^2+^ (Figure 8E). Taken together, our data shown that Na_V_ channel activation by neurotoxins that triggers Ca^2+^ influx is fully mediated by LTCC.

### 2.4. GH3b6 Cell-Based Assay Using Fura-2 Offers a Convenient Model to Characterize Na_V_ Channel Modulators

**Figure 9:**
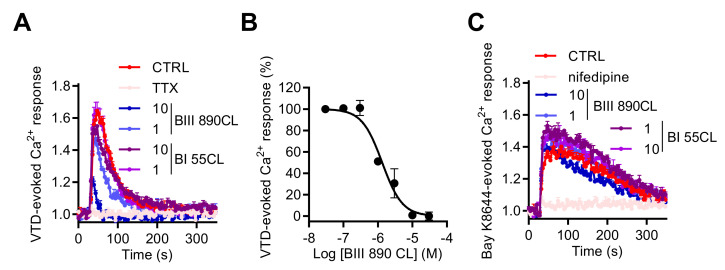
Effects of BIII 890CL on Ca^2+^ responses triggered by VTD or Bay K8644. (**A**) Representative Fura-2 fluorescence kinetic traces illustrating Ca^2+^ response induced by 10 µM of VTD, alone or co-injected with 1 µM of TTX or with 10 or 1 µM of BIII 890 CL or with BI 55CL (10 and 1 µM), used as a negative control. (**B**) Concentration–inhibition relationship of VTD-induced Ca^2+^ responses by BIII 890CL, (IC50 = 1.47 ± 0.18 µM, Hill slope = 1.26 ± 0.23, R^2^ = 0.94). (**C**) Representative Fura-2 fluorescence kinetics traces illustrating Ca^2+^ response induced by 1 µM of Bay K8644, a specific L-type Ca_V_ channel activator, alone or co-injected with 10 µM of nifedipine or with 10 or 1 µM of BIII 890 CL or with BI 55CL (10 and 1 µM), used as a negative control. Data represent the mean ± SEM (*n* = 3) of recordings of two independent experiments.

In order to validate our model as a suitable assay for Na_V_ channel pharmacological studies, we used a novel selective blocker of Site 2 of Na_V_ channels BIII 890 CL (crobenetine) [43,44,45] together with BI 55CL, which is a structurally close analogue of BIII 890 CL but with more than 1000-fold lower potency for Na_V_ channels. When co-injected with VTD (10 µM), 1 or 10 µM of BIII 890 CL blocked the increase of [Ca^2+^]_i_ with a percentage of inhibition of 34.9% ± 0.9% and 74.2% ± 4.2%, respectively, whereas BI 55CL did not significantly modify the Ca^2+^ entry induced by VTD (Figure 9A). The BIII 890 CL inhibition of VTD-evoked Ca^2+^ responses could be fitted by a single site bimolecular equation, giving an IC_50_ value of 1.47 ± 0.18 µM (Figure 9B). Importantly, Ca^2+^ responses induced by Bay K8644, a specific LTCC activator, were not modified by BIII 890 CL nor BI 55 CL whereas 10 µM of nifedipine totally blocked the Bay K8644-evoked Ca^2+^ entry in GH3b6 cells (Figure 9C), demonstrating the selectivity of BIII 890 CL toward Na_V_ channels and the specificity of the Ca^2+^ monitoring when Na_V_ channels are activated by selective neurotoxin. Taken together, the crosstalk between Na_V_ and Ca_V_ channels in GH3b6 cells appears as a new strategy to modulate Na_V_ channels.

## 3. Discussion

All pituitary cells, including GH3 cells and its subclone GH3b6, exhibit membrane excitability and signaling pathways similar to those observed in neurons, because they endogenously express Na_V_ and Ca_V_ channels [22]. Here, we showed that the Na_V_1.3 channel is the predominant subtype expressed at the plasma membrane and endorses the voltage-gated I_Na_ in GH3b6 cells. Various neurotoxins or activators of Na_V_ channels (VTD, BTX, β-PMTX, PbTx2, and deltamethrine) and notably Tf2, a selective neurotoxin of the Na_V_1.3 channel subtype, activate Na_V_ channels in GH3b6 cells, which leads in turn to the elevation of [Ca^2+^]_i_. We determined that this [Ca^2+^]_i_ elevation is due to plasmalemmal Ca^2+^ entry mediated by LTCC, highlighting a crosstalk between Na_V_ channels and Ca_V_ channels in GH3b6 cells. 

GH3b6 cells express TTX-S Na_V_ channel subtype transcripts (*scn2a*, *scn3a*, *scn8a*) commonly expressed in neurons of the central nervous system. It appears that *scn3a* transcript is the most abundant and only the Na_V_1.3 channel subtype was detected at the protein level and at the plasma membrane in these cells. *scn1b* and *scn3b*, encoding β1 and β3 subunits, were also found, in accordance with the high level of Na_V_1.3 expression [46,47]. Since GH3b6 cells are a subclone of GH3 cells, it is not surprising that both share similar Na_v_ channel gene expression profiles except for Na_v_1.1, which was not detected in our model [48,49]. Although we did not detect Na_V_1.2 and Na_V_1.6 channels at the protein level, Na_V_1.2 and Na_V_1.6 channels are likely expressed at very low densities and thus their contribution to I_Na_ could be disregarded. Indeed, the biophysical and pharmacological properties of I_Na_ recorded in GH3b6 cells perfectly match with those of Na_V_1.3 channels. This subtype produces I_Na_ with fast repriming kinetics and recovers rapidly from inactivation, ramp currents, and persistent currents [50,51]. Moreover, Na_V_1.3 channels are blocked by ICA-121431, and were activated at more negative Vm in the presence of the β-scorpion toxin Tf2, both being selective ligands of this Na_V_ channel subtype.

Based on these data, the activation of Na_V_ channels by neurotoxins could lead to [Ca^2+^]_i_ elevation, as previously demonstrated in neuronal cells, through a crosslink between Na_V_ and/or NCX and/or Ca_V_ channels [9,11,14,16,17] but never in endocrine cells. In GH3b6 cells, we found that VTD-induced [Ca^2+^]_i_ elevation was totally blocked by TTX in the nanomolar range, confirming the involvement of TTX-S Na_V_ channels. These Ca^2+^ responses to VTD were only sensitive to Ca_V_ channel inhibitors, such as Cd^2+^ and nifedipine, a selective LTCC inhibitor, which completely blocked the Ca^2+^ entry, with IC_50_ values close to those previously determined by electrophysiology [52,53]. Thus, when VTD induced Na^+^ entry through Na_V_ channels and subsequent membrane depolarization, only LTCC are were and promoted a subsequent large Ca^2+^ influx. This is supported by the Ca_V_ channel gene expression profile, showing LTCC as one of the main abundant Ca_V_ channels in GH3b6 cells (Ca_V_1.2 and Ca_V_1.3). Ca_V_3.1 encoded by *cacna1g* is also expressed in GH3b6 cells at a similar level to *cacna1d*. However, since nifedipine totally blocked VTD-induced Ca^2+^ elevation, T-type Ca_V_ channels certainly do not contribute to these Ca^2+^ entries. Thus, by inducing intracellular Ca^2+^ overload, neurotoxins that activate Na_V_ channels might also alter hormone secretion in endocrine cells.

**Figure 10:**
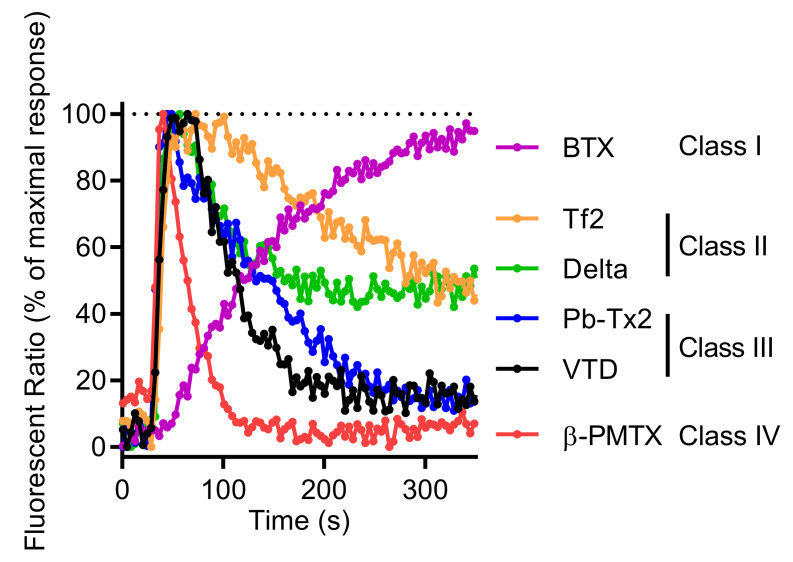
Comparison of representative Fura-2 fluorescence kinetic traces illustrating the Ca^2+^ response induced by 0.6 µM of BTX, 0.5 µM of Tf2, 10 µM of deltamethrin (Delta), 3 µM of Pb-TX2, 10 µM of VTD, and 100 µM of 100 µM β-PMTX. Each kinetic trace was normalized against the maximum of signal, given the 100% of maximum response.

Other neurotoxins activating Na_V_ channels, such as BTX or the β-scorpion toxin Tf2 [32,34], the wasp toxin β-PMTX [31], the marine toxin PbTx-2 [3], and the pyrethroid deltamethrin [54], are also able to increase [Ca^2+^]_i_ in GH3b6 cells. Moreover, the kinetics of Ca^2+^ entry exhibited different patterns according to each type of neurotoxin (Figure 10). Since these Ca^2+^ responses result from membrane depolarization mediated by Na_V_ channels, their distinct kinetics likely reflect the Na_V_ channel gating modification induced by their interaction with these ion channels. For example, although BTX and VTD both bind to Site 2 of open-state Na_V_ channels and block the inactivation process, BTX induced a slow and sustained Ca^2+^ responses, while VTD triggered rapid Ca^2+^ responses (Figure 10). This is probably because BTX permanently maintains Na_V_ channels in the open state, whereas VTD behaves as a partial activator, triggering reversible and rapid alteration of the inactivation process [55,56]. Thus, according to the profile of [Ca^2+^]_i_ elevation kinetics, four types of Na_V_ activator “class” can be distinguished (Figure 10). For class I (BTX), a slow and sustained increase of [Ca^2+^]_i_ time-rate was observed. For class II (deltamethrin and Tf2 toxin), a rapid [Ca^2+^]_i_ increase was followed by a slow decrease. For class III (VTD and PbTx-2), intermediate rapid [Ca^2+^]_i_ elevation kinetics with a peak were seen. Finally, for class IV (β-PMTX), the responses were characterized by a rapid and transient kinetics. Altogether, our model allows the establishment of a “fingerprint” for each class of Na_V_ channel activators.

This strategy could also be useful to characterize new Na_V_ inhibitors. Indeed, we showed that BIII 890 CL, a use-dependent Na_V_ channel blocker [43,44,45], was able to inhibit the VTD-induced Ca^2+^ responses. The absence of effects of BIII 890 CL on Bay K8644-induced Ca^2+^ responses evidenced that BIII 890 CL selectively blocked Na_V_ channels in GH3b6 cells. In addition, the pharmacological profile towards Na_V_ channels has not been described. Our data showed for the first time that BIII 890 CL potently inhibits Na_V_1.3 channels with IC_50_ in the micromolar range. Since only one report has shown a similar IC_50_ (0.6 µM) on Na_V_1.8 channels [44], its selectivity towards the other Na_V_ channel subtypes deserves to be clarified.

Thus, GH3b6 cells appear as an interesting cellular model, which mainly express the Na_V_1.3 channel subtype at the physiological level. This particular Na_V_ channel subtype is now considered as an emerging pharmacological therapeutic target for neurological diseases, such as epilepsy or neuropathic pain, after channel upregulation due to spinal cord injury [57,58,59,60]. The β-scorpion toxin Tf2, which selectively activates the Na_V_1.3 subtype, exhibited the strongest EC_50_ value in the nanomolar range, highlighting the relevance of the use of this toxin for investigating the implication of this ion channel in pain [32]. 

The crosstalk between the Na_V_ and Ca_V_ channel appears to be advantageous for characterizing the pharmacological properties of toxins or drugs. The pharmacological profile of BIII 890 CL, which has been claimed to be a potent blocker of the Na_V_ channel [43], has not been extensively described. In our assay, we rapidly showed that this drug has no effects on LTCC even at high concentrations. The crosstalk between the Na_V_ and Ca_V_ channel has been used in screening test-based assay with SH-SY5Y neuroblastoma cells, which has led to the discovery of potent and selective inhibitors of the Na_V_1.7 channel subtype [17,61], evidencing the interest in using such a strategy. GH3b6 cells could offer a way to follow in parallel, by monitoring [Ca^2+^]i with Fura-2, the possibility to screen for ligands of Na_V_1.3 and LTCC.

## 4. Materials and Methods

### 4.1. Chemicals

BTX and TTX were from Latoxan (Valence, France). VTD was from Santa Cruz Biotechnology (Dallas, TX, USA), Tf2 and pompilitoxin (β-PMTX) were purchased from Smartox Biotechnology (Saint Egrève, France) and PbTx-2 was a gift from Andrea Bourdelais (University of North Carolina Wilmington, Wilmington, NC, USA). BIII 890 CL, such as BI 55 CL, was kindly provided by Boehringer Ingelheim (Biberach an der Riss, Germany) and Bay K8644 was from Alomone (Jerusalem, Israel). All other reagents and solvents, including Fura-2 AM, Pluronic^®^-F127 acid, ICA-121431, SN-6, SEA-0400, deltamethrin, thapsigargin, nifedipine, and cadmium chloride (CdCl_2_), were obtained from Sigma-Aldrich Merck (Saint-Louis, MO, USA) or Thermo Fisher Scientific (Waltham, MA, USA). 

### 4.2. Cell Line

The GH3b6 cells, a subclone of the rat GH3 pituitary cell line [62], were a generous gift of Dr Françoise Macari (IGF, Montpellier, France). GH3b6 cells were cultured from passage 14 until passage 32 at 37 °C/5% CO_2_ in Dubelcco’s Modified Eagle Medium (DMEM)/F12 medium (without L-glutamine, with 15 mM HEPES, with 1.2 g/L NaHCO_3_) (PAN-Biotech, P04-41550) supplemented with 10% fetal bovine serum (Eurobio, CVFSVF00-01), 1 mM L-glutamine (PAN-Biotech, P04-80100), and 1 mM of penicillin/streptomycin (PAN-Biotech, P06-07100) (called DMEM/F12 medium supplemented). GH3b6 cells were routinely cultivated in Flask T75, and when 80% of confluence was reached, split in a ratio of 1/5 in a new Flask T75.DMEM/F12 medium, removed every two or three days, and fresh DMEM/F12 medium supplement added (15 mL for a Flask T75). 

### 4.3. Quantitative Real-Time PCR

First, 100,000 GH3b6 cells per cm^2^ were seeded in multiwell 6 plates in 2 mL of DMEM/F12 medium supplemented by well until 80% confluence. After 3 days of culture, cells were washed in ice-cold PBS and total RNA from GH3b6 cells was extracted using the RNeasy micro kit (Qiagen, Courtaboeuf, France). In total, 1 µg of total RNA was processed for cDNA synthesis using random hexamers and the QuantiTect Reverse Transcription kit (Qiagen). Real-time PCR assays were assessed on a LightCycler 480 Instrument II (Roche, Meylan, France) using Sybr^®^ Select Master Mix (Applied Biosystems^®^), 2.5, 5, and 10 ng of cDNA in duplicate, and gene-specific primers (Supplementary Table S1) previously designed using the Primer3 Software. Amplicon sizes (70–106 bp) and AT% (47–55%) were chosen to allow comparison between the relative expression values obtained for each gene [63]. Differences in transcript expression levels were determined using the cycle threshold method, as described earlier [64]. Amplification specificity was confirmed by one peak–melting curve at the end of the amplification process. Relative quantification of gene expression was normalized to the mean of the expression of two validated housekeeping genes using the 2^−ΔCt^ method, where C_t_ is the threshold cycle: *GAPDH* (glyceraldehyde-3-phosphate dehydrogenase) and *GUSB* (beta-glucuronidase). To perform absolute quantification, synthetic cDNA spanning each PCR amplicon were cloned into pUC57 and sequenced to check if their sequences were identical to those deposited in the GenBank (Genecust, Boynes, France). Absolute quantification of mRNA copies was then carried out using the calibration curve method, using the recombinant double-stranded plasmid DNA molecule determination as already described [65]. The plasmid copy numbers of eight dilutions of pure plasmids were used to establish the calibration curves for each gene. The calibration curves were used to evaluate PCR efficiency, which reached 100% for each gene.

### 4.4. Immunoblot

In total, 100,000 GH3b6 cells per cm^2^ were seeded in multiwell 6 plates and cultivated in 2 mL of DMEM/F12 medium supplemented by well until 80% confluence. After 3 days of culture, cells were washed, scratched in ice-cold PBS, and lysed in 100 µL of RIPA buffer (150 mM Tris-HCl, 50 mM Tris, 12 mM sodium deoxycholate, 0.1% SDS, 1% Triton X-100, pH 8), supplemented with Complete^TM^ Mini Protease Inhibitor cocktail (Roche Applied Science, Laval, Quebec). Lysates were centrifuged for 15 min at 15,000 rpm and supernatants were collected. After protein quantification (Pierce™ BCA Protein Assay Kit), 20–50 µg of either GH3b6 cells or rat brain protein extracts were separated by 8% SDS-polyacrylamide gel electrophoresis and were transferred onto a PVDF membrane 0.45 µM (Thermo Fisher Scientific). The primary antibodies used were anti-pan Na_V_ (rabbit, SP19, ASC-003, Alomone labs, Jerusalem, Israel), anti-Na_V_1.3 (rabbit, ASC-004, Alomone labs or mouse, WH0006328M1, Sigma-Aldrich Merck), and anti-actin used as loading control (mouse, clones AC-203 or AC-74, Sigma-Aldrich Merck). Peroxidase-conjugated secondary antibodies and a Pierce^TM^ ECL-Plus Chemiluminescence kit (Thermo Fisher Scientific) were used before visualization using a LAS-3000 imager (Fujifilm, Tokyo, Japan).

### 4.5. Immunofluorescence Staining

In total, 200,000 cells per cm^2^ were seeded in a Nunc Lab-Tek chamber slide (Thermo Scientific) and cultivated for 3 days and cultivated until 80% confluence. After being washed with ice-cold PBS, the cells were fixed for 10 min with 4% paraformaldehyde, rinsed with PBS, and incubated in blocking solution (10% BSA in PBS) for one hour at room temperature (RT). Immunofluorescence staining was processed, first overnight at 4 °C using the mouse primary antibody anti-Na_V_1.3 (1/50, WH0006328M1, Sigma-Aldrich) and then for one hour at RT using goat anti-mouse IgG (H + L) cross-adsorbed Alexa Fluor 488-conjugated secondary antibody (1/200, Invitrogen). Nuclei were stained with DAPI 10 µg/mL (Molecular probes, Invitrogen). The localization and expression of the targeted proteins were visualized using a Nikon Eclipse TE2000S confocal microscope, and the images were analyzed using the Metamorph^®^ software. Control experiments excluding the primary antibody were performed to verify the specificity of the fluorescence. 

### 4.6. Measurement of Intracellular Ca^2+^

GH3b6 cells were plated at a density of 100,000 cells by well (96 wells black/clear bottom plate) in 100 µL of DMEM/F12 medium supplemented by well. Twenty-four hours after plating, cells were incubated with Fura-2 AM. The Fura-2 AM dye (10 mM, DMSO) was freshly prepared in a Fura-2 buffer composed of Hank’s Balanced Salt Solution (HBSS) supplemented (in mM): 2.5 CaCl_2_, 1 MgCl_2_, 10 HEPES-K, and 0.5% BSA (pH 7.4). Cells were first incubated with Fura-2 AM (4 µM) and Pluronic^®^-F127 acid (0.02%) in Fura-2 buffer for 60 min at RT. After washing, cells were incubated with Fura-2 buffer for 60 min for a complete de-esterification of the dye. The plates were illuminated at 340 and 380 nm excitation wavelengths and the fluorescence emission spectra was recorded at 510 nm using a FlexStation^®^ 3 Benchtop Multi-Mode Microplate Reader. After a 30 s baseline, Na_V_ activators including VTD, BTX, Tf2, PbTx-2, β-PMTX, and deltamethrin and Bay K8644 were automatically injected, and the fluorescence emission spectra were monitored for 320 s at an acquisition frequency of 0.25 Hz. Co-injection of Na_V_ (TTX, BIII 890 CL, or its negative control BI 55 CL) or NCX (SN-6, SEA 0400) or LTCC (nifedipine, CdCl_2_) inhibitors was achieved with activators. All experiments were performed in triplicate at least twice. Data analysis was performed using the SoftMax Pro 5.4.1 software (Molecular Devices, Sunnyvale, CA, USA).

### 4.7. Manual Patch-Clamp Experiments

The manual whole-cell patch-clamp technique [66] was used to record inward Na^+^ currents, using an EPC 10 USB amplifier controlled with PATCHMASTER software (HEKA Elektronik, Lambrecht, Germany). GH3b6 cells were plated at a density of 30,000 cells by glass coverslips and placed in perfusion chamber thermostated at 27 °C. Microelectrodes were prepared by pulling glass capillaries with a resistance of 2.4–2.5 MΩ. The procedure for patch-clamp recordings used in this work followed all recommendations kindly provided by Prof. Stefan H. Heinemann (Friedrich Schiller University of Jena, Jena, Germany) and adapted from previous studies [67,68]. The intracellular solution contained (in mM): 35 CsCl, 80 CsF, 15 NaCl, 10 TEACl, 10 EGTA, and 10 HEPES (pH 7.4, osmolarity 299 mOsm), and the extracellular solution contained (in mM): 140 NaCl, 5 CsCl, 0.2 CdCl2, 2 CaCl2, 1 MgCl2, and 10 HEPES (pH 7.4, osmolarity 290 mOsm). 

### 4.8. High-Throughput Electrophysiology

Automated patch-clamp recordings were performed using the SyncroPatch 384PE from Nanion (München, Germany). Single-hole 384-well recording chips with medium resistance (4.77 ± 0.01 MΩ, *n* = 384) were used for the recordings of HEK-293 cells stably expressing human Na_V_1.3 channel or GH3b6 cells (300,000/mL) in a whole-cell configuration. Pulse generation and data collection were performed with the PatchControl384 v1.5.2 software (Nanion) and the Biomek v1.0 interface (Beckman Coulter). Whole-cell recordings were conducted according to the recommended procedures of Nanion. Cells were stored in a cell hotel reservoir at 10 °C with a shaking speed of 60 rpm. After initiating the experiment, cell catching, sealing, whole-cell formation, buffer exchanges, recording, and data acquisition were all performed sequentially and automatically. The intracellular solution contained (in mM): 10 CsCl, 110 CsF, 10 NaCl, 10 EGTA, and 10 HEPES (pH 7.2, osmolarity 280 mOsm), and the extracellular solution contained (in mM): 140 NaCl, 4 KCl, 2 CaCl2, 1 MgCl2, 5 glucose, and 10 HEPES (pH 7.4, osmolarity 298 mOsm). For GH3b6 cells, 10 µM of nifedipine were added to the external buffer to block LTCC. Whole-cell experiments were performed at a holding potential of −100 mV at room temperature (18–22 °C). Currents were sampled at 20 kHz. Tf2 was diluted in external buffer supplemented with 0.3% BSA. 

### 4.9. Data Analysis

All graphs and statistical analysis were established using GraphPad Prism 7.02 (La Jolla, CA, USA). Data are presented as the mean ± SEM, calculated from at least *n* = 3 replicates and representative of at least 2 or 3 independent experiments. The kinetic traces of Fura-2 fluorescence were plotted as an emission ratio (λ_ex_ 340 nm/λ_ex_ 380 nm). Non-linear analysis with variable slope was used to fit the concentration–response data with the Langmuir–Hill equation. For these analyses, the integration of the fluorescence kinetics (area under curve, AUC) obtained with increasing concentrations were used. Normality of the data distribution was evaluated using the Shapiro–Wilk test before choosing parametric or non-parametric tests. Multiple groups were compared by using a one-way analysis of variance (ANOVA) followed by Tukey post-hoc test or a two-way ANOVA followed by a Bonferroni post-hoc test, when appropriate. Differences between independent groups were assessed by using the non-parametric Mann–Whitney test. Differences with *p* < 0.05 were considered significant (ns: not significant, * for *p* < 0.05, ** for *p* < 0.01, *** for *p* < 0.001, **** for *p* < 0.0001).

## 5. Conclusions

Na^+^ and Ca^2+^ homeostasis is intimately linked in excitable cells thanks to the crosstalk between Na^+^ channels, Ca^2+^ channels, and/or Na^+^/Ca^2+^ exchanger. Here, we demonstrated this crosstalk occurs between the Na_V_1.3 channel subtype and LTCC in the endocrine cell line GH3b6. Thereby, neurotoxins that specifically persistently activate Na_V_ channels induce intracellular Ca^2+^ overload, which could in turn alter hormone secretion. Moreover, GH3b6 cells represent a convenient model for in vitro characterization of neurotoxins targeting Na_V_ channels and particularly those that could be selective for Na_V_1.3 by measuring [Ca^2+^]_i_ levels, thanks to Na_V_–Ca_V_ channels interplay.

## Figures and Tables

**Table 1:** Biophysical parameters of voltage-gated Na^+^ currents recorded in GH3b6 cells.

Condition	Voltage-Dependency of Activation		Voltage-Dependency of Inactivation	
	V_1/2_ (mV)	k (mV)	V_1/2_ (mV)	k (mV)
**Control ^2^**	−12.23 ± 1.34 *n* = 11	6.06 ± 0.20	−53.58 ± 1.66 *n* = 16	5.26 ± 0.17
**Control ^3^**	−8.92 ± 1.01 *n* = 12	6.06 ± 0.20	−56.85 ± 1.54 *n* = 12	5.26 ± 0.17
**+ 1 µM ICA-121431 ^3^**	−11.86 ± 1.05 *n* = 12 (*p* = 0.06)	6.67 ± 0.36 *n* = 12 (*p* < 0.05)	−67.19 ± 1.15 *n* = 12 (*p* < 0.0001)	5.18 ± 0.18 (*p* = 0.72)
**Control ^4^**	−15.07 ± 0.51 *n* = 86	5.78 ± 0.14	−55.69 ± 0.53 *n* = 76	5.86 ± 0.14
**+1 nM Tf2 ^4^**	−26.78 ± 3.24 *n* = 9 (*p* < 0.0001)	5.83 ± 0.52 (*p* = 0.90)	−60.77 ± 1.20 *n* = 24 (*p* < 0.0001)	5.87 ± 0.32 ns, *p* = 0.97

^2^, ^3^ and ^4^ curve fitting parameters of Figure 2, Figure 3 and Figure 4, respectively.

## Data Availability

All the data are contained within the article.

## Supplementary Materials

The following supporting information can be downloaded at: https://www.mdpi.com/article/10.3390/ijms23020827/s1.

## Abbreviations

AUC	area under curve
BTX	batrachotoxin
INa	Na^+^ current
PbTx	brevetoxin
β-PMTX	β-pompidilotoxin
CaV channel	voltage-gated Ca^2+^ channel
[Ca^2+^]i	intracellular Ca^2+^ concentration
GAPDH	glyceraldehyde-3-phosphate dehydrogenase
GUSB	beta-glucuronidase
LTCC	L-type voltage-gated Ca^2+^ channel
NaV channel	voltage-gated Na+ channel
NCX	Na^+^/Ca^2+^ exchanger
TTX	tetrodotoxin
TTX-S	sensitive to tetrodotoxin
TTX-R	resistant to tetrodotoxin;
VTD	veratridine
