# Supplementary material for: Pharmacological Dissection of the Crosstalk between NaV and CaV Channels in GH3b6 Cells
Source: Int J Mol Sci. 2022 Jan 13;23(2):827. doi: 10.3390/ijms23020827 (PMC8775721; doi:10.3390/ijms23020827)

Rhetoré et al., 2021 Supplementary figure S1

Figure S1. Immunoblotting with different antibodies against Na_v channels. Immunoblots with (A) anti-pan-Na_v (ASC-003, Alomone), with (B) anti-Na_v1.3 (ASC-004, Alomone and WH0006328M1, Sigma Aldrich), and with (C) anti-Na_v1.2 (K69/3, Alomone) are showed. The immunodetection of Na_v channels using protein extracts of GH3b6 cells was compared to those of rat brain. Western blotting were performed with two different quantities of protein extracts (20 and 50 µg) which were loaded on 8% SDS-PAGE. Actin was used as a loading control. Pan Nav and anti-Na_v1.3 antibodies allowed the detection of Na_v channels endogenously expressed in GH3b6 cells, but anti-Na_v1.2 antibody did not.

A

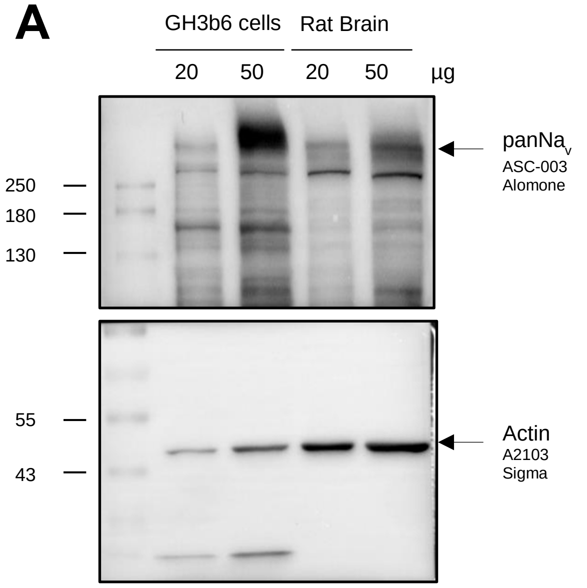

B

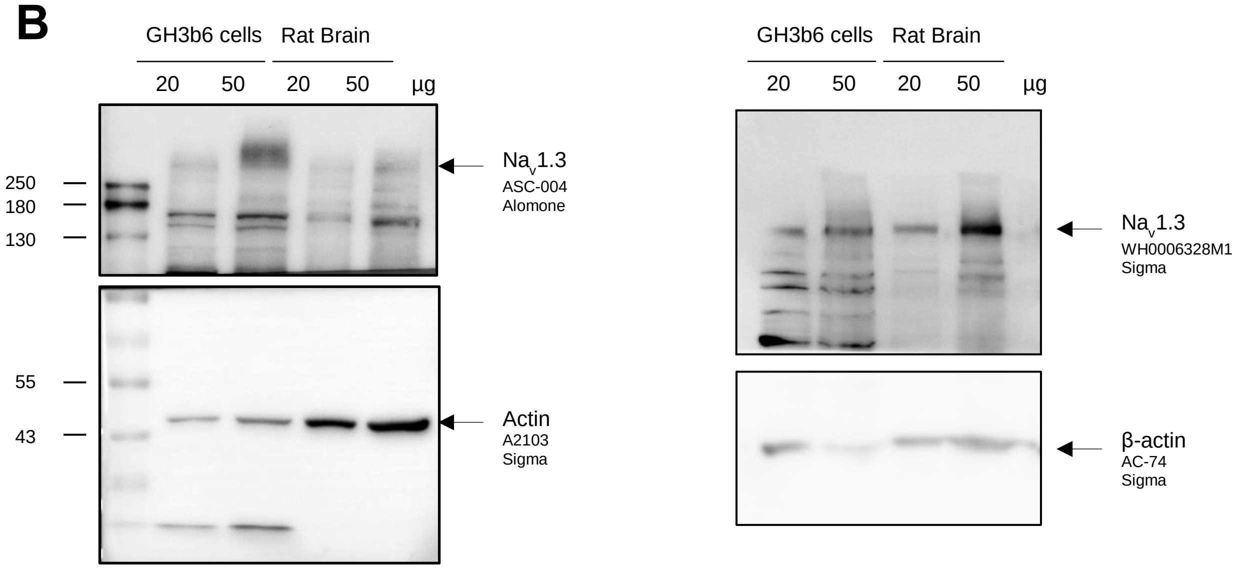

C

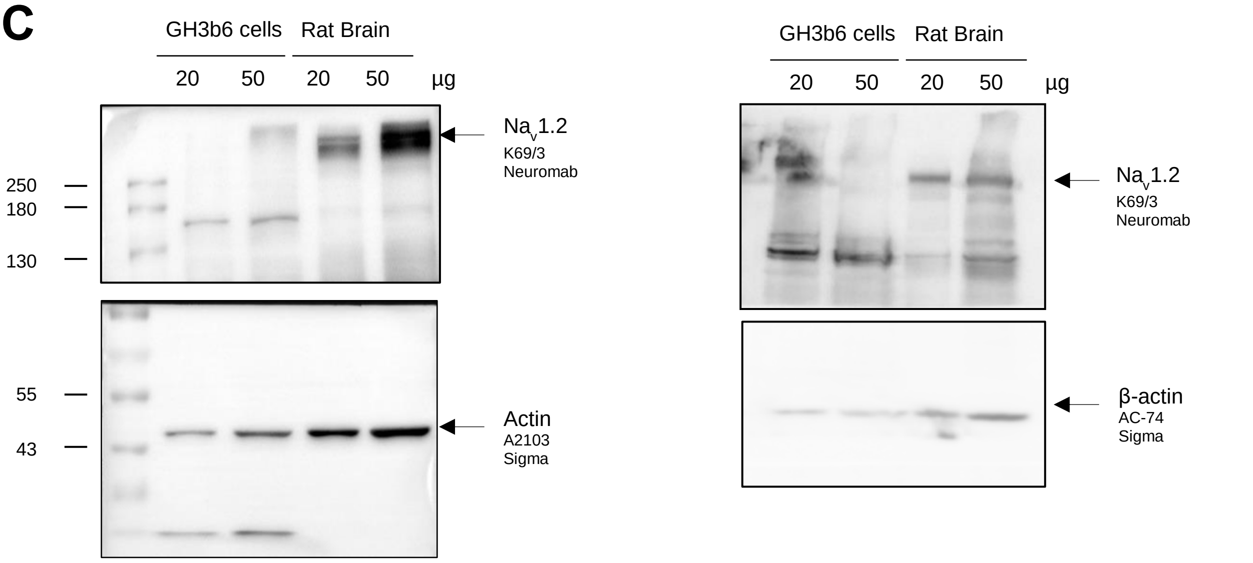

Supplement: Supplementary file 1 [file ijms-23-00827-s001.zip › Figure S1 Rhetore.pdf]
