# Supplementary material for: Pharmacological Dissection of the Crosstalk between NaV and CaV Channels in GH3b6 Cells
Source: Int J Mol Sci. 2022 Jan 13;23(2):827. doi: 10.3390/ijms23020827 (PMC8775721; doi:10.3390/ijms23020827)
Supplement: Supplementary file 1 [file ijms-23-00827-s001.zip › Figure S2 Rhetore.pdf]

Rhetoré et al., 2021 Supplementary figure S2

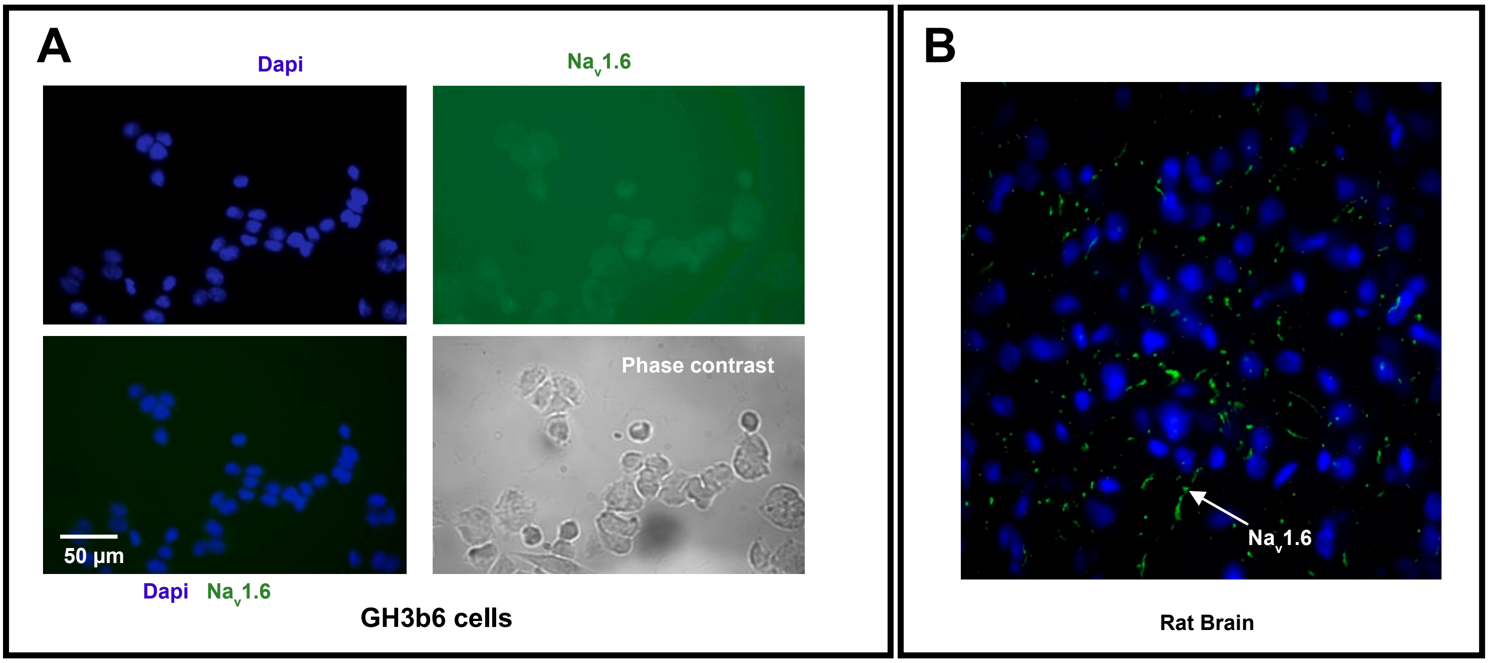

Figure S2. Immunocytochemistry on GH3b6 cells using anti-Na$_v$1.6 channel antibodies. Immunofluorescence labelling shows the detection of Na$_v$1.6 channels (1/50, mouse, clone K87A/70, Neuromab) (green) in rat brain (B) but not at the plasma membrane of GH3b6 cells (A). Nuclei (blue) were stained with DAPI. Arrow shows Na$_v$1.6 expression in rat brain using Alexa Fluor 488 anti-mouse secondary antibody. Original magnification x60.
