# Supplementary material for: Pharmacological Dissection of the Crosstalk between NaV and CaV Channels in GH3b6 Cells
Source: Int J Mol Sci. 2022 Jan 13;23(2):827. doi: 10.3390/ijms23020827 (PMC8775721; doi:10.3390/ijms23020827)
Supplement: Supplementary file 1 [file ijms-23-00827-s001.zip › Figure S3 Rhetore.pdf]

Rhetoré et al., 2021 Supplementary figure S3

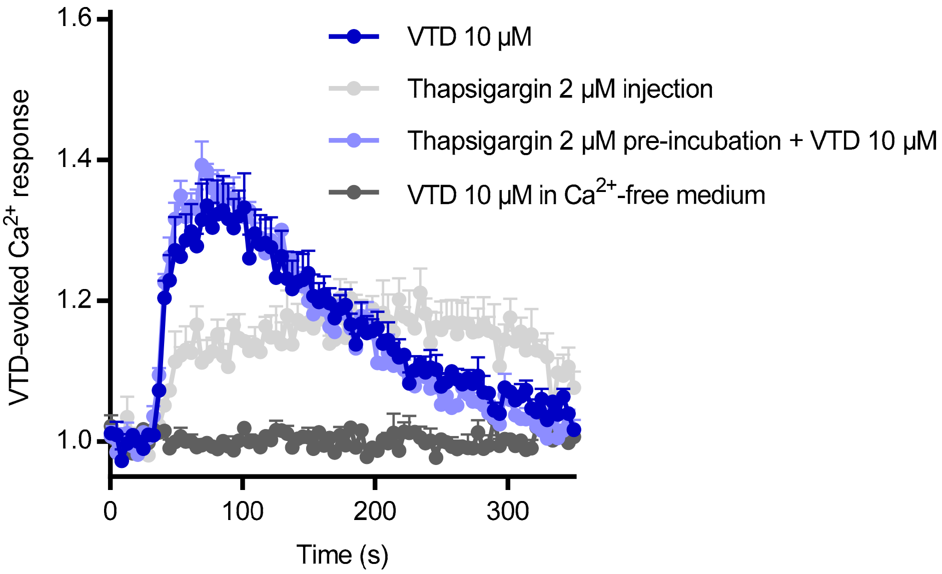

Figure S3. Effects of removal of extracellular Ca^{2+} or Ca^{2+} store depletion on veratridine-induced Ca^{2+} response in GH3b6 cells. Representative kinetics of Fura-2 fluorescence in GH3b6 cells treated with 10 µM of veratridine (VTD) in presence or absence of extracellular Ca^{2+} from the extracellular medium or after a thapsigargin treatment. The VTD-evoked Ca^{2+} response is totally suppressed by Ca^{2+} removal from the extracellular medium but not by a thapsigargin pretreatment (2 µM) which did not impact the VTD-evoked Ca^{2+} response. Thus, VTD induced the elevation of intracellular Ca^{2+} from extracellular Ca^{2+} source.
