# Supplementary material for: Pharmacological Dissection of the Crosstalk between NaV and CaV Channels in GH3b6 Cells
Source: Int J Mol Sci. 2022 Jan 13;23(2):827. doi: 10.3390/ijms23020827 (PMC8775721; doi:10.3390/ijms23020827)
Supplement: Supplementary file 1 [file ijms-23-00827-s001.zip › Table S1- Rethore 2021.pdf]

gene name	GenBank accession number	protein name	forward primer (5'-3')	reverse primer (5'-3')
Scn1a	NM_030875	Na_V1.1	gttccgacatcgccagtt	catctcagtttcagtagttgttcca
Scn2a	NM_012647	Na_V1.2	tggtgtccctggttggag	ccttatttctgtctcagtagttgtgc
Scn3a	NM_013119	Na_V1.3	gcaccgtccattctaaccat	tttagcttcttgcataagaattgc
Scn4a	NM_013178	Na_V1.4	ggcactgtctcgatttgagg	ttcatgatggaggggatagc
Scn5a	NM_013125	Na_V1.5	tgccaccaatgccttgta	catgatgagcatgctaaagagc
Scn8a	NM_019266	Na_V1.6	ggaagttttccatcatgaatcag	gctgttatgtcgggagagga
Scn9a	NM_133289	Na_V1.7	cagcagatgttagaccgactca	actcgtgaactcagcagcag
Scn10a	NM_017247	Na_V1.8	agaggaccccaagggaca	tggtggttttcacacttttgg
Scn11a	NM_019265	Na_V1.9	cagaggacgatgcctctaaaa	ttctgggacagtcgtttggt
Scn1b	NM_017288	$Na_V\beta$1	gcggagatggtgtactgctac	tcggaagtaatggccaggtat
Scn2b	NM_012877	$Na_V\beta$2	tggacttaccaggagtgtagca	ccagcttcaggttgatgatct
Scn3b	NM_139097	$Na_V\beta$3	ctgatacctttgcgagtcactg	catcatgatttccgagaccac
Ssn4b	NM_001008880	$Na_V\beta$4	catcctgaagaagaccagagaga	agcagggcctcacacttttt
Slc8a1	NM_019268	NCX1	tccgattccctctaccgtaa	gtcagtggctgcttgtcatc
Slc8a2	NM_078619	NCX2	tgtgcactacgggatgctt	cgtctccactatcttgacct
Slc8a3	NM_078620	NCX3	gggagctggagtttaagaatga	gaagaaattctcttgcctttcg
Cacna1s	NM_053873	Ca_V1.1	gcgtcgtggagtggaaac	gggagctggagtttaagaatga
Cacna1c	NM_012517	Ca_V1.2	tggctcacagaagtgcaaga	agcatttctgccgtgaaaag
Cacna1d	NM_017298	Ca_V1.3	ccatgctcactgtgttccag	ctcccatcctatcgcatcat
Cacna1a	NM_012918	Ca_V2.1	ctgctttgaagaggggacag	ctgacatttggtcccgttg
Cacna1b	NM_147141	Ca_V2.2	taagcgcatcaccgaatg	ggccaggacaatacagttgg
Cacna1e	NM_019294	Ca_V2.3	taccgcgcctggatagac	gctgatgttcccgagttttt
Cacna1g	NM_031601	Ca_V3.1	catctacttcattcttctcatcatcg	aactgcgtggcaatcacc
Cacna1h	NM_153814	Ca_V3.2	acggatactctgcagacagga	gctctgtgtagtctgggatgc
Cacna1i	NM_020084	Ca_V3.3	tctgcctcaatgttgtcacc	aagggctgtctctagggatgt
Gusb	NM_017015.2	Gus	ctctggtggccttacctgat	cagactcaggtgttgtcatcg
Gapdh	NM_017008.3	Gapdh	tgggaagctggtcatcaac	gcatcaccccatttgatgtt

Table S1 : Primers used for RT-qPCR
